# Building bilateral global research capacity among students and faculty through initiation of a medical student research grant in Ghana

**DOI:** 10.1002/ijgo.15847

**Published:** 2024-08-09

**Authors:** Dhanalakshmi Thiyagarajan, Rebecca Ibine, Mercy Nuamah, Emma Lawrence

**Affiliations:** ^1^ Department of Obstetrics and Gynecology University of Michigan Ann Arbor USA; ^2^ Department of Obstetrics and Gynecology Family Health Medical School Accra Ghana; ^3^ Department of Community Health Family Health Medical School Accra Ghana

**Keywords:** bilateral capacity‐building, children's health, faculty development, global health, medical student research, research capacity‐building, women's health

## Abstract

This global grant program provides the opportunity for students to develop meaningful projects and translate them into academic success, while building bilateral faculty research capacity.

## 1 INTRODUCTION

Publications from the Global South comprise less than 10% of worldwide research articles.^1^ Key barriers, including experience, mentorship, and funding, limit low‐ and middle‐income country (LMIC) researchers from producing research articles.^2^ Interventions addressing these barriers from career inception can encourage more LMIC medical researchers to conduct meaningful research. Research capacity‐building is an important aspect of medical education.^3^ Global collaboration for high‐income countries' researchers promotes opportunities to ethically engage in global research.^1^ We outline the key principles, development, and outcomes of a bilateral medical student grant program aimed to build early research capacity through global research collaboration.

## 2 METHODS

**FIGURE 1:**
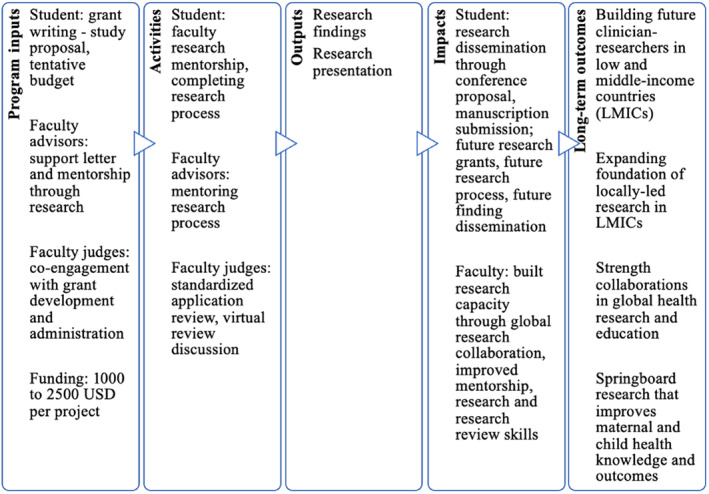
Logic model for interventional impact.

A medical student research grant program was established in 2022 between the Family Health Medical School (FHMS) in Ghana and the University of Michigan (UM) in the US. The main objectives of the research grant program are to: (1) alleviate financial barriers by considering projects limited by funding; (2) encourage Ghanaian medical students to learn the process of applying to a research grant; (3) strengthen medical education and build research capacity among Ghanaian and American faculty by co‐engaging in grant development and administration, mentorship, and research dissemination; and (4) foster global research collaboration to springboard research that improves maternal and child health knowledge and outcomes in relation to the Sustainable Development Goals, 2030 (Figure 1).^4^

Given FHMS's partnership with UM's Department of Obstetrics and Gynecology, donor funding was leveraged from previous capacity‐building success.^5^ All procedures, policies, and documentation were co‐developed by faculty members from FHMS and UM. The grant program supports Ghanaian medical students' mentored women's or children's health research. Students' applications consist of student and faculty advisor information, a letter of support from the advisor, and research proposal (title, background, objectives, study design, significance, timeline, personal impact, itemized budget). Using standardized forms, grants are reviewed by faculty and senior trainees from Ghana and UM on scientific merit, significance, feasibility, and personal commitment to research. Five grants are awarded yearly, each providing US$1000–2500. Student recipients receive mentorship in their research area from the FHMS faculty, as well as co‐mentorship from the UM faculty. Faculty advisors have support from their research chair to build their skillset. Research results are disseminated as oral or poster presentations at local and international conferences, and manuscripts are submitted for publications in a peer‐reviewed scientific journal.

## 3 RESULTS

The inaugural grant call was sent to 120 FHMS medical students via their school mailing list. Ten eligible submissions were received: seven on women's health topics and three on children's health topics. Six faculty and senior trainees from Ghana and six from UM with expertise in maternal or child health were invited to be grant reviewers. Each application was reviewed by one Ghanaian and one UM reviewer. Virtually, a review committee of FHMS and UM faculty discussed final scores. Five grants were awarded, three women's health‐focused and two children's health‐focused, for a total of US$8600. Awarded topic areas included cervical cancer screening, dysmenorrhea, hypertensive disorders of pregnancy, childhood diarrhea, and parasitic worm infection. All five grant awardees completed the mentored research process from research question to design to data collection and analysis. The results were disseminated in a book of abstracts and poster presentations at FHMS graduation in the presence of FHMS and UM faculty and sponsors. Three manuscripts are submitted to the peer‐reviewed *International Journal of Health and Medical Sciences*, and two manuscripts are under submission to other peer‐reviewed journals.

Students' motivation to participate included: (1) desire to identify the root cause of health outcomes; (2) build critical‐thinking and innovation skills; and (3) gain support for future research careers. Advisors' motivation to participate included the development of (1) the next generation of researchers; and (2) their mentorship and research skills. After program completion, all students and advisors viewed the program favorably and would participate again. Students believed they accomplished their personal goals for participation and are utilizing this experience to pursue future research career opportunities. Advisors believed they made a positive impact on the students' research career by demonstrating to the students their learnt ability to be primary investigator from idea conception to research dissemination.

## 4 DISCUSSION

Utilizing funding and mentoring resources, Ghanaian medical students successfully experienced the research process. Given the importance of research dissemination, co‐mentorship extended to poster presentations at a shared research day between FHMS and UM, and preparation of manuscripts. Additionally, the faculty members serving as committee members, evaluators or advisors built capacity by developing their mentorship and research skills. There is opportunity for improvement by formalizing the mentorship that faculty members receive, evaluating the program's long‐term impact, and receiving more administrative and financial support. The program's aim is that students' learned skills can be translated into future research success by replicating this process in the future for other important research questions. This research grant program can be implemented at other global partner institutions using a similar protocol to build collaborative global research capacity.

## AUTHOR CONTRIBUTIONS

DT contributed towards conduct, data analysis, and manuscript writing. RI contributed towards design, planning, conduct, and manuscript writing. MN contributed towards design, planning, conduct, and manuscript writing. EL contributed design, planning, conduct, data analysis, and manuscript writing. All authors read and approved the final manuscript for submission.

## FUNDING INFORMATION

The grant is provided by a private donor, Elaine Schweitzer, and the Schweitzer Family Foundation.

## CONFLICT OF INTEREST STATEMENT

The authors have no conflicts of interest.

## Data Availability

Data sharing is not applicable to this article as no new data were created or analyzed in this study.
